# The Relationship Between Breast Milk Adipocytokine Levels and Anthropometric Measurements of SGA Infants in the First Year of Life

**DOI:** 10.3390/nu18213569

**Published:** 2026-10-28

**Authors:** Zeynep Yılmaz Öztorun, Fatih Kardaş, Nihal Hatipoğlu, Durmus Ayan, Şerife Buket Bozkurt Polat, Meda Kondolot

**Affiliations:** 1 Social Pediatrics Unit, Department of Pediatrics, Atatürk Sanatoryum Training and Research Hospital, 06290 Ankara, Türkiye; 2 Department of Pediatric Metabolism, Faculty of Medicine, Erciyes University, 38039 Kayseri, Türkiye; fkardas@erciyes.edu.tr; 3 Department of Pediatric Endocrinology, Faculty of Medicine, Erciyes University, 38039 Kayseri, Türkiye; nihalhatipoglu@yahoo.com; 4 Department of Medical Biochemistry, Faculty of Medicine, Niğde Ömer Halisdemir University, 51240 Niğde, Türkiye; durmusayan@hotmail.com (D.A.); buketbozkurt@yahoo.com (Ş.B.B.P.); 5 Department of Medical Biochemistry, Dr Abdurrahman Yurtaslan Ankara Oncology Training and Research Hospital, 06200 Ankara, Türkiye; 6 Social Pediatrics Unit, Department of Pediatrics, University of Health Sciences, Ankara Etlik City Hospital, 06170 Ankara, Türkiye; medakondolot@gmail.com

**Keywords:** human milk, SGA, adiponectin, visfatin, obestatin, nesfatin-1

## Abstract

**Background/Objective:** Adipocytokines play a role in infant growth and metabolic programming. This study was conducted to investigate the relationship between obestatin, nesfatin-1, visfatin, and adiponectin levels in breast milk and serum of Small for Gestational Age (SGA) infants and their anthropometric measurements up to one year of age. **Methods:** Thirty SGA infants and forty AGA (appropriate for gestational age) infants were included in study, and their anthropometric measurements were taken in the 1st, 2nd, 3rd, 4th, 6th, 9th, and 12th months of life. Nesfatin-1, adiponectin, obestatin, visfatin levels were examined in breast milk samples and infant serum at 1st and 3rd months using ELISA. **Results:** Serum and breast milk adiponectin levels at the 1st month were significantly associated with the longitudinal change in head circumference. Breast milk adiponectin at the 1st month was negatively associated with the longitudinal change in head circumference (*p* = 0.04), while serum adiponectin was positively associated with head circumference change in the first year (*p* = 0.007). Serum visfatin levels in the 1st month were negatively associated with longitudinal triceps skinfold (TSF) changes (*p* = 0.04), and there was no relation between nesfatin-1 or obestatin levels in breast milk or serum and changes in the anthropometric indices of infants up to one year of age. **Conclusions:** Breast milk and serum adiponectin may have an effect on head circumference development up to 1 year of age. Specifically, serum visfatin influenced changes in the infant’s TSF over time and may lead to the fat mass accumulation. Further studies with longer follow-up periods are needed to examine the effect of adipocytokines on the growth of SGA infants.

## 1. Introduction

Breast milk has a unique composition, containing bioactive substances, macronutrients, micronutrients, and hormones that promote optimal growth and development during infancy and provide protection against the development of obesity and metabolic syndrome in later life [1,2]. Hormones found in breast milk, which play various roles in regulating appetite, have been defined as adipocytokines [3]. It has been demonstrated that adipocytokines regulate weight gain, fat mass, and lean body mass in infants during the early postpartum period, and long-term effects on metabolic programming have been reported [4]. Breast milk and infant serum are biologically distinct compartments: milk adipocytokines represent maternal-origin exposure, whereas infant serum levels reflect the infant’s own circulating hormonal status. Since milk-borne hormones may be absorbed by the immature neonatal gut and influence appetite and growth regulation [5,6,7], evaluating both compartments may provide complementary insights into postnatal growth [8,9]. There are limited number of studies examining the relationship between infants’ anthropometric measurements and the various adipocytokines which have different anorexogenic or obesogenic effects.

Adiponectin is known to be synthesized in adipose tissue, where it stimulates food intake and reduces energy expenditure. At high concentrations, it has been shown to contribute to a slower rate of gastric emptying [9]. Additionally, high levels of adiponectin in breastfed infants may contribute to a slower progression along the growth curve [10].

Nesfatin-1, which is synthesized in adipose tissue, as well as the central and peripheral nervous systems, has been shown to have anorexogenic effects by reducing food intake. It has been proposed that it contributes to a reduction in adipose tissue by inducing a feeling of satiety [11]. However, studies examining the relationship between nesfatin-1 in breast milk and anthropometric measurements have yielded conflicting results [11,12].

Obestatin may contribute to a reduction in appetite through its ghrelin-antagonist effect, and its levels in breast milk decrease over time after birth [13]. Therefore, it is of interest to determine how changes in its levels over time might affect the infant’s growth and changes in anthropometric measurements.

Studies have shown that serum visfatin levels are significantly elevated in infants with intrauterine growth restriction and very low birth weight [14] and studies examining the effect of breast milk visfatin on infant growth are limited.

Small for Gestational Age (SGA) infants have a high risk of developing obesity and metabolic disorders later in life [15]. It is thought that this condition may be related to the metabolic adaptation experienced by SGA infants during the intrauterine period and their growth pattern in the postnatal period. The effects of adipocytokines on weight gain and the regulation of fat mass and lean body mass have been demonstrated, and these effects may differ between SGA and Appropriate for Gestational Age (AGA) infants. The aim of our study is to investigate the relationship between obestatin, nesfatin-1, visfatin, and adiponectin levels in breast milk and infant serum, and the anthropometric measurements of SGA infants up to 1 year of age. The findings from this study may contribute to our understanding of how the adipocytokine profile in breast milk of SGA infants affects their growth during infancy and may serve as a guide for future research in this area.

## 2. Materials and Methods

### 2.1. Study Design and Participants

This prospective observational study was conducted at the Pediatrics Outpatient Clinic of Niğde Ömer Halisdemir Training and Research Hospital (NOHU) between September 2022 and February 2024.

The sample size was calculated using the G*Power program (version 3.1) under the following conditionsan: an alpha level of 0.05, a target statistical power of 0.95, and an effect size of 1.095. Cohen’s d was calculated as the mean difference divided by the pooled standard deviation [72.4/√((86.5^2^ + 35.4^2^)/2) ≈ 72.4/66.1], yielding d = 1.095 [8]. To our knowledge, Filibeli’s study was the only published study reporting this specific outcome in comparable groups, which necessitated using these values as our baseline. Additionally, their study involved the longitudinal monitoring of SGA and AGA infants and analyzed adipocytokines—such as nesfatin and adiponectin—similar to those examined in our own study. Based on the power analysis, the sample size was calculated as minimum of 23 infants in the SGA group and 23 in the AGA group.

A total of 85 infants—38 SGA (small for gestational age) and 47 AGA (appropriate for gestational age) were included in the study. Among the SGA infants, 2 developed a food allergy, and 1 developed immune hemolytic anemia, 2 developed hypothyroidism, 1 developed bronchopneumonia, while 2 infants were excluded from the study because they did not continue breastfeeding during the first 3 months. Among the AGA infants, 2 were excluded because their families did not consent to the collection of serum samples in the 3rd month, 1 due to the occurrence of convulsions, 1 due to a diagnosis of hypothyroidism, and 3 because they were no longer being fed with breast milk.

The study was continued with 70 infants: 30 infants in the SGA group and 40 infants in the control group (appropriate for gestational age, AGA). Infants with a birth weight below the 10th percentile for their gestational age were defined as SGA. The control group consisted of breastfed AGA infants whose birth weight was between the 10th and 97th percentiles for gestational age. Infants in both the SGA and control groups who were breastfed for at least the first 3 months were included in the study. Both the SGA and control groups were fed supplementary formula alongside breast milk after the 3rd month, and both groups started complementary feeding after the 6th month. The exclusion criteria were as follows: (1) having a congenital anomaly, (2) having had an intrauterine infection, (3) a history of asphyxia at birth, (4) preterm birth, (5) multiple pregnancy, (6) having a chronic illness, (7) being fed formula in addition to breast milk during the first three months after birth. Additionally, the exclusion criteria for the control group included the development of a chronic disease during the study period, hospitalization, or feeding with formula during the first 3 months.

Blood samples were collected from infants in the SGA and control groups at the 1st and 3rd month postnatal follow-ups to assess nesfatin-1, adiponectin, obestatin, and visfatin levels; breast milk samples were also collected from their mothers when the babies reached 1 and 3 months of age. Anthropometric measurements including body weight, height, head circumference, and skinfold thickness (triceps muscle) were taken from all infants at the 1st, 2nd, 3rd, 4th, 6th, 9th, and 12th months of age. The relationship between breast milk and serum adipocytokines and anthropometric measurements was examined.

#### Ethics Approval

This study was approved by the Ethics Committee for Noninterventional Clinical Research of NOHU, Faculty of Medicine (approval number: 2022/79, 11 August 2022) and was conducted in accordance with the principles of the Declaration of Helsinki. Written informed consent was obtained from the parents prior to study participation. The study was supported by the Scientific Research Project Unit of Erciyes University (project number: TDK-2022-12374).

### 2.2. Measurements

#### 2.2.1. Anthropometric Measurements

Height was measured in the supine position on a standard infant measuring board (±0.1 cm) while head circumference was measured using a non-stretchable tape measure placed from the most prominent point on the forehead to the most prominent point at the back of the head, with a precision of 0.1 cm. Skinfold thickness was measured in the triceps region using a skinfold caliper and the weight for length (WFL) calculation was performed by dividing the infant’s measured body weight by the ideal body weight for that height and multiplying the result by 100.

#### 2.2.2. Biochemical Analysis

Breast milk and infant serum samples were collected between 8:00 a.m. and 11:00 a.m., 2 h after the completion of the previous breastfeeding session. Before collecting breast milk, mothers were instructed on the manual milk expression method, and 10 mL of breast milk was collected. Breast milk samples were centrifuged at 4000 rpm for 10 min and then stored by freezing at −80 °C in Eppendorf tubes. Five milliliters of venous blood samples were collected from the infants. After centrifugation at 4000 rpm for 10 min, serum samples were aliquoted and stored at −80 °C until analysis. Before the analysis, the frozen samples were thawed gradually at room temperature and mixed gently.

Serum nesfatin-1, adiponectin, obestatin, and visfatin, and concentrations were determined using commercially available human sandwich enzyme-linked immunosorbent assay (ELISA) kits (ELK Biotechnology Co., Ltd., Wuhan, China) according to the manufacturer’s instructions. The following ELISA kits were used: Human Adiponectin (Cat. No. ELK1223), Human Obestatin (Cat. No. ELK5409), Human Visfatin (Cat. No. ELK1725), and Human Nesfatin-1 (Cat. No. ELK2828).

The absorbance of each well was measured at 450 nm using a microplate reader (Epoch™ 2 Microplate Spectrophotometer, BioTek Instruments Inc., Winooski, VT, USA), and biomarker concentrations were calculated from the standard calibration curves generated for each analyte using the standards supplied with the respective kits.

Serum nesfatin-1, adiponectin, obestatin, and visfatin levels were analyzed using commercial ELISA kits according to the manufacturer’s instructions. The measurement range of the visfatin ELISA kit was 0.16–10 ng/mL, with an analytical sensitivity of 0.059 ng/mL. Obestatin was measured with a commercial Human Obestatin ELISA kit (Cat. No. ELK5409, ELK Biotechnology, Wuhan, China) following the manufacturer’s instructions. The standard curve ranged from 78.13 to 5000 pg/mL, and the manufacturer-reported sensitivity was 25.1 pg/mL. All milk samples were diluted 1:20 in the kit’s sample diluent before analysis. Final concentrations were calculated by multiplying each reading by the dilution factor of 20. Consequently, values above 5000 pg/mL in the manuscript are dilution-corrected concentrations and do not represent extrapolation beyond the calibration range. With this dilution, the effective quantification range of the assay in milk was 1562.6 to 100,000 pg/mL. All samples yielded readings within the standard curve. No values fell below the lower limit of quantification or above the upper limit, and no samples required re-analysis at a different dilution.

The measurement range of the nesfatin-1 ELISA kit was 781.25–50,000 pg/mL, with an analytical sensitivity of 234.2 pg/mL, while the adiponectin ELISA kit was reported to have a measurement range of 0.16–10 ng/mL and an analytical sensitivity of 0.069 ng/mL. For all kits, the intra-assay coefficient of variation (CV) reported by the manufacturer was below 8%, and the inter-assay coefficient of variation was below 10%.

#### 2.2.3. Statistical Analysis

Data were analyzed using SPSS, version 25 (SPSS, Chicago, IL, USA). For normally distributed numerical data, mean and standard deviation (mean ± SD) values were used. To compare independent numerical variables, the Student’s *t*-test was used if the data were normally distributed, while the Mann-Whitney U test was used if they were not normally distributed. Correlation analyse was performed using the Spearman correlation method due to the non-normal distribution of the data. Longitudinal anthropometric measurements were analyzed using linear mixed-effects models to account for the correlation between repeated measurements obtained from the same infant. Infant ID was specified as a random effect, while time, the SGA/AGA group, and the time*group interaction were included as fixed effects. Potential confounding variables, including birth weight, birth length, birth head circumference, gender, and maternal gestational weight gain, were included in the models as appropriate. The longitudinal associations of breast milk and serum nesfatin-1, adiponectin, obestatin, and visfatin concentrations with weight, length, and head circumference, triceps skinfold (TSF), and WFL were also evaluated using linear mixed effects models. Regression coefficients (β), standard errors, 95% confidence intervals, and *p*-values were reported. Numerous adipocytokine and outcome associations were tested and the possibility of false positivitesy due to multiple testing may emerge. A two-sided *p*-value < 0.05 was considered statistically significant.

## 3. Results

### 3.1. Sociodemographic Characteristics and Anthropometric Measurements

Of the SGA infants 50% were male, while the remainder were female, and the distribution of delivery modes was as follows: 63.3% cesarean section (C/S) and 36.7% normal vaginal delivery. The mean birth weight of the SGA infants was 2377.7 ± 53.9 g (mean ± SD), while that of the AGA infants were 3235.1 ± 291.2 g. The gestational weight gain of the SGA infants’ mothers was 11.1 ± 2.3, while that of the AGA infants’ mothers were 11.2 ± 3.8 kg. The weights, heights, head circumferences, of SGA infants at 1st, 2nd, 3rd, 4th, 6th, 9th, and 12th months were found to be significantly lower than those of AGA infants (*p* < 0.05). Additionally, TSF measurements in the SGA infants were lower than those in AGA infants only at 1st month and 9th months (*p* = 0.007 and *p* = 0.03, respectively); no significant differences in TSF were found in the other months (Table 1).

### 3.2. Findings from Biochemical Analysis

Adipocytokine levels in breast milk and serum of SGA and AGA infants are presented in Table 2. Serum visfatin levels in SGA infants at the 1st month were found to be higher than those in the AGA infants (*p* = 0.004); no significant differences were observed between the groups in terms of other serum adipocytokine levels at either the 1st or 3rd month (*p* > 0.05) (Table 2). It was detected that nesfatin and obestatin levels in breast milk decreased over time, while visfatin and adiponectin levels increase over time. In the serum nesfatin and obestatin levels increased, while visfatin and adiponectin levels decreased in SGA infants by the third month.

### 3.3. Correlation Between Serum Visfatin and Anthropometric Measurements in SGA and AGA Infants

A significant moderate positive correlation was found between serum visfatin levels in the first month and weight in the 4th month (r = 0.143, *p* = 0.02) and as well as between serum visfatin levels in the first month and WFHs in months 2, 3, and 4 (*p* = 0.03; Table 2), in SGA infants. There was a negative correlation between serum visfatin levels and triceps thickness at months 6, 9 and 12, as well as between the serum visfatin levels of AGA infants and head circumference at 12 months (*p* < 0.05) (Table 3).

#### 3.3.1. Longitudinal Changes in Anthropometric Measurements Using Linear Mixed Effect Models

Time significantly affected weight, length, and head circumference during the first year (all *p* < 0.05). The time*group interaction was not significant for weight (*p* = 0.867) or length (*p =* 0.109), but it was significant for head circumference (*p* = 0.001), indicating different longitudinal growth patterns between SGA and AGA infants. This finding demonstrates that head circumference development differs significantly over time between SGA and AGA infants. Maternal gestational weight gain was significantly and negatively associated with infant weight (β = −62.27; *p* = 0.02) (Table 4). Triceps skinfold (TSF) changed significantly over time (F = 396.660; *p* = 0.001), and the time*group interaction was significant (F = 2.581; *p* = 0.018), indicating different longitudinal changes between SGA and AGA infants. WFL did not show a significant overall change over time (F = 1.245; *p* = 0.283), but the time*group interaction was significant (F = 2.610; *p* = 0.017), indicating different longitudinal changes between the groups. Gender and maternal gestational weight gain were not significantly associated with either outcome (*p* > 0.05) (Table 4).

#### 3.3.2. Longitudinal Associations Between Breast Milk/Serum Adipocytokines and Anthropometric Measurements During First Year

Anthropometric measurements increased significantly over time during the first year. Serum and breast milk adiponectin levels at 1 month of age were significantly associated with the longitudinal change in head circumference (*p* < 0.05). In contrast, while breast milk adiponectin at the 1st month was negatively associated with the longitudinal change in head circumference, serum adiponectin was positively associated. Serum visfatin levels at the 1st month were significantly negatively associated with longitudinal TSF changes (*p* < 0.05), whereas none of the breast milk or serum adipocytokines showed a significant association with longitudinal WFL changes (*p* > 0.05). Nesfatin-1 and obestatin were not significantly associated with longitudinal changes in weight, length, and head circumference (Table 5).

## 4. Discussion

In our prospective study, we investigated the relationship between breast milk and serum adipocytokine levels and weight, height, head circumference, triceps skinfold thickness, and WFL. While the measurement of serum adiponectin in the first month was positively associated with the longitudinal change in infants’ head circumference up to one year of age, breast milk adiponectin was negatively associated with the longitudinal change in head circumference. It was determined that serum visfatin at one month of age influences the change in triceps skinfold thickness during the first year of life. In SGA infants, serum visfatin levels in the 1st month showed a significant positively correlation with WFH and weight.

The differing effects of adipocytokines on metabolic regulation, whether anorexogenic or obesogenic may explain the opposite correlations observed with anthropometric measurements.

It has been reported that adiponectin is 40 times more abundant than leptin in breast milk and acts as an appetite regulating hormone, and various studies have examined the effects of adiponectin on infant growth [16]. It was found that high levels of breast milk adiponectin at birth in preterm infants born at less than 33 weeks were associated with a higher length Z-score and a lower head circumference in the 1st month. Adiponectin levels in breast milk decreased during the first month after birth, affecting the total amount of adiponectin ingested through breast milk [17]. In a study using the RIA method, a negative correlation was found between breast milk adiponectin levels in the 3rd months and 3rd month head circumferences and WFL values in the 3rd month, and this negative correlation was observed in infants of both mothers with gestational diabetes and healthy mothers [18]. In the study by Nunes et al., it was shown that in SGA infants, as well as in infants of mothers with gestational diabetes and gestational hypertension, breast milk adiponectin levels decreased as the transition from colostrum to mature milk occured, and there was no association between breast milk adiponectin and weight gain in infants during the first month [19]. Additionally, the absence of an association between breast milk adiponectin and height measurements in SGA infants suggests that height catch-up in SGA infants may be more closely related to genetic potential and nutrition, and that height growth in SGA infants may be more influenced by factors such as the growth hormone-insulin like growth factor-1 (GH-IGF-1) axis rather than adiponectin [20].

In a study involving term SGA and AGA newborns aged 24h, a positive correlation was found between serum adiponectin levels and head circumference [21]. In a study of SGA preterm infants, serum adiponectin levels increased from birth to the 3rd week, and a positive correlation was found between serum adiponectin and head circumference, weight, and height [22]. Bozzola et al. reported that serum adiponectin levels in SGA infants increase during the first month and subsequently decrease, but that serum adiponectin shows no correlation with head circumference, weight, or height during the first 12 months [23].

The literature presents conflicting results regarding the relationship between serum adiponectin and anthropometric measurements in SGA children, with some studies reporting a negative association and others indicating a positive one [15,24]. The findings by Kamoda et al., who showed that serum adiponectin is positively correlated with head circumference during the neonatal period, were consistent with the relationship observed in our study, that is, between serum adiponectin and the increase in head circumference in AGA infants [21].

In our study, it was determined that breast milk adiponectin was negatively associated with changes in head circumference during the first year of life, whereas serum adiponectin was positively associated with changes in head circumference. Head circumference growth may be positively affected as the adiponectin level in the infant’s own circulation increases. The finding of an inverse relationship between head circumference and serum adiponectin versus breast milk adiponectin may indicate that infant serum and breast milk adiponectin may act through different biological pathways or receptor mechanisms. Additionally, the possible existence of significant associations between adiponectin and head circumference has been demonstrated. There is a need for future studies that will present the data on this subject more clearly.

Nesfatin-1 is thought to reduce the risk of obesity [25]. The decrease in its levels in breast milk over time and the increase in serum levels over time were consistent with the literature [12]. However, the number of studies on this topic was limited. In a study examining the relationship between nesfatin-1 levels and anthropometric measurements in SGA infants, a negative correlation was found only between nesfatin-1 in breast milk in the first month and head circumference [12]. When comparing nesfatin-1 levels in breast milk between two groups where one group had a high weight for age Z-score and the other had a low weight-for-age Z-score, no difference in nesfatin-1 levels was detected, and no significant association was found between breast milk nesfatin-1 levels and the infants’ anthropometric measurements [26]. There are a limited number of studies examining the effect of breast milk and serum nesfatin-1 levels on infant growth in the SGA group. Studies have not examined the long-term effect of nesfatin-1 on longitudinal changes in the anthropometric measurements of infants. In the present study, it was found that nesfatin-1 levels were not associated with changes in anthropometric measurements during the first year of infancy. Thus, a greater number of studies regarding this adipocytokine are needed.

Knowledge regarding the physiological role of obestatin, a peptide known to function as a ghrelin antagonist during infancy, is limited [27]. It has been determined that obestatin is present in breast milk, and its concentration is higher in colostrum than in mature milk [28]. In our study, no significant association was found between breast milk and serum obestatin and anthropometric indices in the first year. Additionally, it has been reported that decreased plasma ghrelin levels are associated with insulin resistance. In a study involving adolescents, serum obestatin levels were positively correlated with BMI [29]. Considering the studies conducted, ghrelin changes with the increase in body fat mass and has effects on growth. It was hypothesized that this mechanism might provide protection against childhood obesity by preventing a rapid increase in fat mass. Since no studies have examined the relationship between obestatin, both in breast milk and in serum, and growth parameters, longitudinal studies beginning in infancy could contribute to clearer findings regarding this adipocytokine.

It has been reported that visfatin levels in breast milk and serum change over time after birth, and these changes have been linked to intrauterine growth restriction, preterm birth, preeclampsia, and gestational diabetes [30]. In the study by Bienertoa-Vasku et al., breast milk visfatin concentration increased in AGA infants from birth through the first month and then gradually decreased; however, the changes in breast milk visfatin levels during the first six months were not considered statistically significant [31]. In very low birth weight preterm infants, visfatin levels in breast milk gradually increased from the first week to the the fifth week [32]. Infants with higher colostrum visfatin levels experience more pronounced weight loss in the first few days of life, whereas those with lower colostrum visfatin levels experience less weight loss [31]. In the present study, we found that visfatin levels in breast milk increased by the third month in SGA infants, while serum visfatin levels decreased. In AGA infants, it was detected that both breast milk and infant serum visfatin levels increased by the third month. Studies have shown that changes in visfatin levels may vary in different directions over time.

This study is significant because it is the first to examine the relationship between visfatin levels in breast milk and anthropometric measurements in SGA infants. However, changes in breast milk visfatin over time did not appear to have any effect on the baby’s growth parameters in the present study.

Visfatin, synthesized from visceral adipose tissue, regulates energy balance and, inflammation [33]. It has been reported that visfatin exhibits pro-inflammatory properties, and high serum levels of this adipocytokine are associated with obesity and metabolic syndrome [34]. Mol et al. detected that a negative correlation was found between serum visfatin levels and weight and head circumference Z scores at 5 weeks of age in preterm infants [32]. In a study examining the correlation between visfatin levels in umbilical cord blood and birth anthropometric indices in SGA infants; no significant correlation was found between umbilical cord blood visfatin levels and birth weight, length, or head circumference. Thus, it was reported that serum visfatin levels did not have a significant effect on anthropometric indices at birth [35]. In our study, the fact that serum visfatin levels at the first month in AGA infants were negatively correlated only with head circumference at 12 months limited the ability to draw meaningful conclusions regarding the impact of this adipocytokine on neurodevelopment. Different etiologies, such as the mother’s body mass index and gestational age, may have influenced the relationship between visfatin and growth parameters at birth. In another study examining the correlation between serum visfatin levels and body mass index (BMI) and wrist circumference in obese children, a positive correlation was found between serum visfatin levels and BMI and wrist circumference [36]. In the study by Simoes et al., which included obese and healthy children aged 6–10 years, it was stated that TSF increased as serum visfatin levels increased in healthy children, and that visfatin may be associated with central obesity [37]. In our study, the gradual decrease in serum visfatin was associated with the expansion of the TSF up to the age of 1 year. The positive correlation found between serum visfatin and WFH in SGA infants at certain months suggests that this adipocytokine may influence increases in body mass and play a role in infant growth. Our study also demonstrated that decreased serum visfatin levels in AGA infants inversely affected triceps skinfold at certain months. In our analysis covering the period up to 12 months of age, TSF in AGA infants increased gradually. In other words, TSF in AGA infants increased as serum visfatin levels decreased. Considering this together with other studies related to serum visfatin, it can be hypothesized that it may have an effect on fat mass accumulation.

The present study includes several strengths, including as prospective follow-up, repeated anthropometry, paired milk and serum sampling, and an AGA comparison group.

The limitations of our study are the collection of breast milk and serum adipocytokine measurements only in the first and third months, the inability to examine breast milk and serum measurements in the later months of life (the 6th, 9th, and 12th months), the unknown obesity status of the mothers, and the exclusion of the mothers’ serum adipocytokine levels from the examination. The lack of follow-up for patients after the 12th month was considered another limitation. Data on maternal BMI, maternal age, gestational diabetes status, and smoking during pregnancy, later formula and complementary feeding quantification were not included in the study. The relationship between breast milk and infant serum adipocytokine levels was not assessed in the present study; therefore, the extent to which milk-derived adipocytokines contribute to infant circulating levels could not be determined. Future studies evaluating paired milk and serum samples are needed to clarify this relationship. Given that our power analysis relied on baseline data from a single, limited cohort, the observed large effect size (1.095) may be subject to inflation; consequently, these findings must be interpreted cautiously and replicated in future investigations with larger sample sizes. Because of the generalizability is limited, larger multicenter studies are needed. These factors have been acknowledged as limitations of the current study. Although the number of cases included in the study was sufficient to achieve statistical significance according to the power analysis, studies with a larger number of patients may be needed to evaluate the correlation relationships with greater statistical power.

## 5. Conclusions

Serum and breast milk adiponectin may affect head circumference development in the first year of life. Serum visfatin levels had an effect on TSF expansion up to the first year of life. In SGA infants, changes in serum visfatin levels over time may have an impact on fat mass increase. In conclusion, further studies are needed to examine the growth outcomes in SGA infants and the relationship between growth and adipocytokines.

## Figures and Tables

**Table 1:** Comparison of anthropometric parameters in SGA and AGA infants.

Months	Anthropometric Parameters	Group		
		SGA (*n*: 30)	AGA (*n*: 40)	*p* *
1st month	Weight (g) Mean ± SD	3554.2 ± 84.1	4496.4 ± 72.3	0.0001 **
2nd month		4520.0 ± 103.9	5456.3 ± 97.8	0.0001 **
3rd month		5477.0 ± 141.1	6415.3 ± 149.8	0.0001 **
4th month		6210.0 ± 133.1	7022.5 ± 152.0	0.0001 **
6th month		7220.0 ± 126.4	7955.0 ± 148.9	0.001 **
9th month		8373.3 ± 166.9	9117.5 ± 157.3	0.002 **
12th month		9310.0 ± 174.8	10,110.0 ± 181.1	0.003 **
1st month	Length Mean ± SD	50.7 ± 0.3	53.2 ± 0.2	0.0001 **
2nd month		54.0 ± 0.3	56.7 ± 0.3	0.0001 **
3rd month		57.4 ± 0.4	60.3 ± 0.4	0.0001 **
4th month		59.8 ± 0.4	62.4 ± 0.4	0.0001 **
6th month		63.9 ± 0.5	66.1 ± 0.3	0.0001 **
9th month		68.5 ± 0.4	70.3 ± 0.3	0.001 **
12th month		72.9 ± 0.3	74.2 ± 0.3	0.01 *
1st month	Head circumference (cm) Mean ± SD	35.9 ± 0.2	37.3 ± 0.2	0.0001 **
2nd month		37.4 ± 0.1	38.9 ± 0.1	0.0001 **
3rd month		39.1 ± 0.2	40.4 ± 0.2	0.0001 **
4th month		40.4 ± 0.2	41.5 ± 0.2	0.0001 **
6th month		42.3 ± 0.1	43.2 ± 0.1	0.001 *
9th month		43.9 ± 0.2	44.8 ± 0.2	0.0001 **
12th month		45.5 ± 0.1	46.0 ± 0.1	0.04 *
1st month	Triceps skinfold (mm) Mean ± SD	14.2 ± 0.3	15.1 ± 0.2	0.007 **
2nd month		15.2 ± 0.2	16.3 ± 0.6	0.10
3rd month		15.9 ± 0.1	16.4 ± 0.2	0.06
4th month		16.5 ± 0.2	16.9 ± 0.1	0.11
6th month		17.1 ± 0.1	17.4 ± 0.2	0.18
9th month		17.6 ± 0.1	18.0 ± 0.1	0.03 *
12th month		18.4 ± 0.1	18.7 ± 0.1	0.09
1st month	Weight for length (WFL)	101.8 ± 2.1	110.5 ± 1.4	0.001 **
2nd month		105.3 ± 1.7	109.0 ± 1.6	0.12
3rd month		105.2 ± 2.1	107.4 ± 1.9	0.43
4th month		106.2 ± 1.8	108.1 ± 1.6	0.44
6th month		104.8 ± 1.3	107.3 ± 1.5	0.26
9th month		105.1 ± 1.4	109.1 ± 1.6	0.08
12th month		104.6 ± 1.4	110.1 ± 1.7	0.02 *

* *p* < 0.05, ** *p* < 0.01, *t*-test.

**Table 2:** Comparison of breast milk and serum adipocytokines in SGA and AGA infants.

Months	Breast Milk Adipocytokine Mean ± SD	Group		*p* *
		SGA (*n*: 30)	AGA (*n*: 40)	
1st month	Breast milk nesfatin-1	50,867.1 ± 5117.4	48,067.8 ± 4023.7	0.72
	Breast milk adiponectin	0.4 ± 0.01	0.2 ± 0.01	0.26
	Breast milk obestatin	52,144.6 ± 9208.8	32,055.7 ± 5277.1	0.08
	Breast milk visfatin	0.6 ± 0.05	0.6 ± 0.04	0.97
3rd month	Breast milk nesfatin-1	32,477.3 ± 4748.6	32,212.5 ± 3298.5	0.96
	Breast milk adiponectin	0.6 ± 0.2	0.4 ± 0.1	0.42
	Breast milk obestatin	4206.9 ± 530.2	3678.4 ± 437.3	0.44
	Breast milk visfatin	1.0 ± 0.07	1.1 ± 0.04	0.86
	Serum Adipocytokine Mean ± SD	SGA	AGA	*p*
1st month	Serum nesfatin-1	16,658.9 ± 1621.8	17,073.4 ± 1082.2	0.58
	Serum adiponectin	4.5 ± 0.4	4.1 ± 0.3	0.41
	Serum obestatin	7255.9 ± 527.3	7827.2 ± 413.9	0.49
	Serum visfatin	3.9 ± 0.3	2.9 ± 0.2	0.004 **
3rd month	Serum nesfatin-1	28,729.2 ± 4186.8	28,648.9 ± 2906.5	0.50
	Serum adiponectin	2.3 ± 0.3	1.9 ± 0.2	0.41
	Serum obestatin	10,953.4 ± 831.9	11,842.8 ± 794.4	0.53
	Serum visfatin	3.4 ± 0.3	3.3 ± 0.2	0.69

Data were presented as mean ± SD. *t*-test. * *p* < 0.05, ** *p* < 0.01.

**Table 3:** Correlation between serum visfatin and anthropometric measurements in SGA and AGA infants.

		*r*	*p*
1st month serum visfatin of SGA infants (ng/mL)	2nd month WFH	0.359	0.04 *
	3rd month WFH	0.362	0.03 *
	4th month WFH	0.371	0.04 *
	4th month weight (g)	0.431	0.017 *
1st month serum visfatin of AGA infants (ng/mL)	6th month triceps thickness	−0.324	0.04 *
	9th month triceps thickness	−0.351	0.03 *
	12th month triceps thickness	−0.361	0.02 *
	12th month head circumference (cm)	−0.317	0.04 *

* *p* < 0.05, Spearman correlation analysis.

**Table 4:** Longitudinal changes in anthropometric measurements according to SGA/AGA group using linear mixed-effects models.

Weight (g)	F	β	SE	95% CI	*p*
Fixed Effect					
Time (Month)	499.094	-	-	-	0.001 **
Group (SGA/AGA)	20.444	-	-	-	0.001 **
Time*Group	0.418	-	-	-	0.87
Birth weight (g)	1.440	0.2059	0.1716	−0.1364–0.5481	0.23
Gender (F/M)	1.421	−191.87	160.93	−512.89–129.16	0.23
Maternal gestational weight gain (kg)	5.673	−62.27	26.14	−114.42–−10.12	0.02 *
Length (cm)					
Time (Month)	1043.389	-	-	-	0.001 **
Group (SGA/AGA)	22.698	-	-	-	0.001 **
Time*Group	1.746	-	-	-	0.11
Birth length (cm)	0.024	−0.019	0.122	−0.262–0.225	0.88
Gender (F/M)	0.869	−0.374	0.401	−1.173–0.425	0.35
Maternal gestational weight gain (kg)	3.555	−0.122	0.065	−0.252–0.007	0.06
Head circumference (cm)					
Time (Month)	790.605	-	-	-	0.001 **
Group (SGA/AGA)	22.175	-	-	-	0.001 **
Time*Group	4.323	-	-	-	0.001 **
Birth head circumference (cm)	0.031	0.018	0.103	−0.187–0.224	0.86
Gender (F/M)	0.967	−0.209	0.212	−0.631–0.214	0.30
Maternal gestational weight gain (kg)	0.609	−0.026	0.034	−0.094–0.041	0.44
Triceps skinfold (mm)					
Time (Month)	396.660	-	-	-	0.001 **
Group (SGA/AGA)	1.201	1.215	0.540	0.139–2.290	0.28
Time*Group	2.581	-	-	-	0.018 **
Gender (F/M)	0.093	0.142	0.465	−0.789–1.074	0.76
Maternal gestational weight gain (kg)	1.421	-	-	-	0.18
Weight for length (WFL)					
Time (Month)	1.245	-	-	-	0.28
Group (SGA/AGA)	3.208	-	-	-	0.08
Time*Group	2.610	-	-	-	0.017 **
Gender (F/M)	0.152	−0.080	0.207	−0.493–0.332	0.70
Maternal gestational weight gain (kg)	0.858	-	-	-	0.60

Infant ID was included as a random effect, F: Linear Mixed-Effects Model, β: Regression coefficient, SE: Standard error, CI: Confidence interval, * *p* < 0.05, ** *p* < 0.01.

**Table 5:** Longitudinal associations between breast milk/serum adipocytokines and anthropometric measurements during the first year.

Weight (g)						TSF (mm)				
Adipocytokine	Source/Time	β	SE	95% CI	*p*	Source/Time	β	SE	95% CI	*p*
Nesfatin-1	Serum, 1 month	0.0046	0.0146	−0.0246–0.0338	0.75	Serum, 1st	0.000006	0.000028	−0.000048–0.000060	0.83
	Serum, 3 month	0.0018	0.0056	−0.0094–0.0131	0.74	Serum, 3rd	−0.000007	0.000010	−0.000027–0.000013	0.48
	Breast milk, 1 month	0.0062	0.0047	−0.0033–0.0157	0.20	Breast milk, 1st	0.000007	0.000008	−0.000008–0.000022	0.34
	Breast milk, 3 month	0.0013	0.0057	−0.0101–0.0128	0.82	Breast milk, 3rd	−0.000002	0.000009	−0.000020–0.000015	0.78
Adiponectin	Serum, 1 month	81.77	58.54	−35.56–199.09	0.17	Serum, 1st	−0.186	0.106	−0.395–0.022	0.08
	Serum, 3 month	−16.60	72.46	−161.84–128.64	0.82	Serum, 3rd	0.089	0.137	−0.179–0.357	0.51
	Breast milk, 1 month	−198.76	300.44	−800.94–403.43	0.51	Breast milk, 1st	−0.755	0.563	−1.859–0.349	0.18
	Breast milk, 3 month	−58.86	106.01	−271.34–153.16	0.58	Breast milk, 3rd	−0.272	0.190	−0.644–0.101	0.15
Obestatin	Serum, 1 month	0.0130	0.0370	−0.0611–0.0873	0.73	Serum, 1st	0.000113	0.000072	−0.000029–0.000254	0.12
	Serum, 3 month	−0.0038	0.0231	−0.0499–0.0424	0.87	Serum, 3rd	0.000051	0.000041	−0.000029–0.000132	0.21
	Breast milk, 1 month	−0.0011	0.0026	−0.0064–0.0042	0.69	Breast milk, 1st	–0.000004	0.000005	−0.000013–0.000006	0.42
	Breast milk, 3 month	−0.0082	0.0468	−0.1020–0.0857	0.86	Breast milk, 3rd	–0.000029	0.000072	−0.000171–0.000113	0.69
Visfatin	Serum, 1 month	17.75	61.88	−106.28–141.77	0.78	Serum, 1st	−0.232	0.117	−0.460–−0.003	0.04 *
	Serum, 3 month	7.79	70.77	−134.06–149.64	0.91	Serum, 3rd	0.031	0.138	−0.240–0.302	0.82
	Breast milk, 1 month	107.40	390.47	−675.24–890.04	0.78	Breast milk, 1st	−0.307	0.758	−1.793–1.179	0.69
	Breast milk, 3 month	120.62	446.34	−773.998–1015.23	0.79	Breast milk, 3rd	−0.799	0.697	−2.164–0.567	0.25
**Length (cm)**						**WFL**				
**Adipocytokine**	**Source/Time**	**β**	**SE**	**95% CI**	***p***	**Source/Time**	**β**	**SE**	**95% CI**	***p***
Nesfatin-1	Serum, 1 month	0.000052	0.000033	−0.000014–0.000119	0.12	Serum, 1st	−0.000021	0.000012	−0.000045–0.000003	0.08
	Serum, 3 month	−0.000003	0.000013	−0.000028–0.000023	0.84	Serum, 3rd	0.000005	0.000004	−0.000004–0.000014	0.28
	Breast milk, 1 month	0.000012	0.000011	−0.000010–0.000033	0.28	Breast milk, 1st	−0.000002	0.000003	−0.000009–0.000005	0.58
	Breast milk, 3 month	0.000006	0.000013	−0.000028–0.000023	0.84	Breast milk, 3rd	−0.000002	0.000004	−0.000010–0.000005	0.55
Adiponectin	Serum, 1 month	0.2159	0.1335	−0.0511–0.4829	0.11	Serum, 1st	−0.003	0.049	−0.098–0.093	0.95
	Serum, 3 month	0.1240	0.1653	−0.2065–0.4552	0.46	Serum, 3rd	−0.085	0.061	−0.204–0.034	0.16
	Breast milk, 1 month	−0.1987	0.6853	−1.5691–1.1717	0.77	Breast milk, 1st	−0.350	0.252	−0.845–0.144	0.17
	Breast milk, 3 month	0.0373	0.2418	−0.4462–0.5208	0.88	Breast milk, 3rd	−0.064	0.086	−0.232–0.105	0.46
Obestatin	Serum, 1 month	0.000077	0.000084	−0.000092–0.000246	0.36	Serum, 1st	0.000004	0.000033	−0.000060–0.000069	0.89
	Serum, 3 month	−0.000036	0.000053	−0.000141–0.000069	0.49	Serum, 3rd	−0.000007	0.000019	−0.000044–0.000030	0.71
	Breast milk, 1 month	0.000005	0.000006	−0.000007–0.000017	0.41	Breast milk, 1st	−0.000003	0.000002	−0.000007–0.000001	0.19
	Breast milk, 3 month	−0.000030	0.000107	−0.000243–0.000184	0.78	Breast milk, 3rd	−0.000014	0.000032	−0.000078–0.000049	0.66
Visfatin	Serum, 1 month	0.008947	0.1412	−0.2733–0.2912	0.95	Serum, 1st	0.041	0.054	−0.064–0.146	0.45
	Serum, 3 month	0.03484	0.1614	−0.2880–0.3576	0.83	Serum, 3rd	−0.070	0.061	−0.191–0.050	0.25
	Breast milk, 1 month	−0.1832	0.8907	−1.9642–1.5978	0.84	Breast milk, 1st	−0.188	0.339	−0.853–0.477	0.58
	Breast milk, 3 month	−0.8744	1.0182	−2.9102–1.1614	0.39	Breast milk, 3rd	0.289	0.313	−0.325–0.902	0.36
**Head Circumference**										
**Adipocytokine**	**Source/Time**	**β**	**SE**	**95% CI**	***p***					
Nesfatin-1	Serum, 1 month	0.000012	0.000017	−0.000022–0.000046	0.48					
	Serum, 3 month	−0.000002	0.000006	−0.000015–0.000011	0.77					
	Breast milk, 1 month	0.000008	0.000005	−0.000002–0.000019	0.12					
	Breast milk, 3 month	−0.000008	0.000007	−0.000021–0.000005	0.23					
Adiponectin	Serum, 1 month	0.182	0.065	0.052–0.311	0.007 *					
	Serum, 3 month	−0.071	0.081	−0.232–0.091	0.39					
	Breast milk, 1 month	−0.713	0.341	−1.396–0.030	0.04 *					
	Breast milk, 3 month	0.054	0.120	−0.186–0.294	0.65					
Obestatin	Serum, 1 month	−0.000006	0.000041	−0.00088–0.000076	0.89					
	Serum, 3 month	0.000036	0.000026	−0.000016–0.000088	0.17					
	Breast milk, 1 month	0.000001	0.000003	−0.000005–0.000007	0.68					
	Breast milk, 3 month	0.000058	0.000056	−0.000055–0.000170	0.31					
Visfatin	Serum, 1 month	−0.068	0.076	−0.221–0.084	0.37					
	Serum, 3 month	−0.024	0.080	−0.185–0.136	0.76					
	Breast milk, 1 month	−0.242	0.446	−1.135–0.651	0.59					
	Breast milk, 3 month	−0.466	0.470	−1.407–0.474	0.33					

Model: Linear mixed-effects model; infant ID was included as a random effect, F: Linear Mixed-Effects Model, β: Regression coefficient, SE: Standard error, CI: Confidence interval, TSF: Triceps skinfold, WFL: Weight for length, * *p* < 0.05.

## Data Availability

The data presented in this study are available on reasonable request from the corresponding author. The data are not publicly available due to privacy and ethical restrictions.

## Abbreviations

The following abbreviations are used in this manuscript:

SGA	Small for gestational age
AGA	Appropriate for gestational age
WFL	Weight for length
BMI	Body mass index
IGF	Insulin like growth factor
TSF	Triceps Skinfold
